# Immobilization of Enzyme Electrochemical Biosensors and Their Application to Food Bioprocess Monitoring

**DOI:** 10.3390/bios13090886

**Published:** 2023-09-17

**Authors:** Ganchao Sun, Xiaobo Wei, Dianping Zhang, Liben Huang, Huiyan Liu, Haitian Fang

**Affiliations:** 1 School of Food Science and Engineering, Ningxia University, Yinchuan 750021, China; sgc1755530869@163.com (G.S.); weixiaobo@nxu.edu.cn (X.W.); 2 School of Mechanical Engineering, Ningxia University, Yinchuan 750021, China; zhangdp@nxu.edu.cn; 3 Huichuan Technology (Zhuhai) Co., Ltd., Zhuhai 519060, China; fhtlhy@163.com

**Keywords:** electrochemical biosensors, enzyme immobilization, nanomaterials, food analysis, process monitoring

## Abstract

Electrochemical biosensors based on immobilized enzymes are among the most popular and commercially successful biosensors. The literature in this field suggests that modification of electrodes with nanomaterials is an excellent method for enzyme immobilization, which can greatly improve the stability and sensitivity of the sensor. However, the poor stability, weak reproducibility, and limited lifetime of the enzyme itself still limit the requirements for the development of enzyme electrochemical biosensors for food production process monitoring. Therefore, constructing sensing technologies based on enzyme electrochemical biosensors remains a great challenge. This article outlines the construction principles of four generations of enzyme electrochemical biosensors and discusses the applications of single-enzyme systems, multi-enzyme systems, and nano-enzyme systems developed based on these principles. The article further describes methods to improve enzyme immobilization by combining different types of nanomaterials such as metals and their oxides, graphene-related materials, metal–organic frameworks, carbon nanotubes, and conducting polymers. In addition, the article highlights the challenges and future trends of enzyme electrochemical biosensors, providing theoretical support and future perspectives for further research and development of high-performance enzyme chemical biosensors.

## 1. Introduction

More than a half-century has passed since Clark and Lyons introduced the enzyme glucose biosensor in 1962 [1], and this important area has been a huge focus of research activity. Compared with traditional analytical methods such as gas and liquid chromatography [2,3], enzyme-based electrochemical biosensors have significant advantages—for example, high selectivity, high sensitivity, relatively fast and simple analytical procedures, and small size of the measurement unit as well as high throughput and portability [4,5]. Based on these findings, enzyme electrochemical biosensors play an important role in the fields of food processing monitoring and quality management, environmental pollution monitoring and analysis, fermentation process control, and biomedical and drug sensing [6,7]. In recent years, many enzyme electrochemical biosensors were conceived, developed, and commercialized as user-friendly and time-saving analytical methods. By selecting different enzymes as the immobilized and sensitive bioelements that recognize the analytes to construct the corresponding biosensors, they can provide reliable output signals quantitatively correlated with the concentration of a specific analyte for the determination of a variety of substances, such as glucose [8], lactose [9], and ethanol [10], among others. And it has proven to be an innovative technique for qualitative and quantitative analysis of various target substrates in a wide range of applications [11].

However, enzyme electrochemical biosensors still face several challenges, such as their insufficient reusability and vulnerability to environmental impacts, which must be addressed to increase their commercial value and efficiency of use. Previous studies have focused on electrochemical biosensors designed for single-enzyme systems. These studies have proved valuable in improving the sensitivity, reproducibility, and stability of sensor devices. However, further efforts are needed to overcome the limitations associated with enzyme electrochemical biosensors. Yet, most enzymes do not expend or produce Electrochemically Active Substances (EAS) as part of the catalysis process, and therefore electrochemical sensors are not able to directly record enzymatic catalytic reactions. As a result, the number of enzymes available for the development of biosensors, as well as the range of compounds that can be targeted, remains limited [12]. To address this problem, scientists have attempted to add several enzymes to the biorecognition element of a biosensor to form a cascade reaction for detecting the product of an enzymatic reaction [11]. The application of multi-enzyme systems in biosensors based on this formation not only aims to realize the detection of single (multiple) analytes, but also improves the performance of biosensors. With the development of technology, it has been found that some of the nanomaterials (1-100nm) have enzyme-like properties and are known as the next generation of artificial enzymes (nano enzymes). Highly stable and inexpensive compared to natural enzymes, these artificial enzymes are favored by many scientists for their ability to mimic the architecture, function, and activity of naturally occurring enzymes, covering their kinetics and mechanisms [13,14]. However, due to the excellent specificity and sensitivity of immobilized enzyme biosensors to specific analytes [15], as well as the problems of nano-enzymes in terms of selectivity, poor biocompatibility, toxicity, and low enzyme activity criteria [16], natural enzyme biosensors are still the dominant research direction at present. In summary, we can see that in the past, scientists have reviewed various aspects of single-enzyme systems, multi-enzyme systems, and nano-enzyme systems in the field of food analysis as well as biosensing, but there is no specific article that can cover all three systems at the same time in a systematic manner.

An important factor to consider when fabricating enzyme electrochemical biosensors is how to immobilize the enzyme on the surface of the working electrode. One of the challenges in enzyme immobilization is that it is difficult to exchange electrons with the electrode interface due to the depth of the active center; in addition, the shape of the enzyme may change after immobilization on the surface of the working electrode [17]; and another challenge is how to prevent denaturation and inactivation of the enzyme, which will ultimately prolong the service life of the biosensor. Therefore, immobilization of the enzyme on a compatible substrate is essential to maintain the stability of the enzyme’s catalytic properties and biological activity. Furthermore, when enzymes are immobilized for use in electrochemical sensors, the issue of enzyme orientation is an important factor affecting the performance of the sensors due to its effect on the generation and transfer of electrons to the electrode surface. In order to optimize the enzyme activity, the orientation of the enzyme should be precisely controlled during the experiment. In the past, rational surface modification techniques based on the understanding of the interactions between enzymes and specific modifiers have been developed to control the orientation of redox enzymes for improved direct electron transfer (DET) type bioelectrocatalysis. In addition, nanomaterials with suitable pore sizes to balance enzyme adsorption, electron transfer, and mass transfer are also expected to be suitable for high-performance DET-type bioelectrocatalysis [18].

During the past years, electrochemical biosensor research has been primarily focused on nanomaterial-modified electrodes because these materials show special physio-chemical characteristics at the nanoscale (e.g., metal nanoparticles, graphene-associated materials, metal-organic frameworks, carbon nanotubes, conductive polymers, etc.) [19], which can be utilized to increase the fundamental analytical properties of biosensors, such as sensitivity, the limit of detection, linear detection range, stability, etc. [20]. In addition, nanomaterials are characterized by high surface-to-volume ratios, high electrical conductivity, magnetism, and catalytic activity, which ensure a significant increase in sensor-sensitive surfaces, allow for easy immobilization of receptors through covalent and noncovalent bonds, provide more efficient sites for enzyme immobilization, and permit the construction of biosensor devices with improved analytical properties, which are essential for biosensors and other biotechnological assays in which interactions with biomaterials are interactions are critical.

In this article, we focus on summarizing the construction principles of enzyme electrochemical biosensors and the recent research advances in single-enzyme systems, multi-enzyme systems, and nano-enzymatic systems for food bioprocess monitoring. Subsequently, the expanded applications of various types of nanomaterials for enzyme immobilization in electrochemical enzyme biosensors in recent years are presented, and the structures and properties of the corresponding sensing platforms are discussed. Finally, we discuss some of the challenges and emerging trends in the design of enzyme-based biosensors. This is expected to provide strong theoretical support for subsequent research development.

## 2. Enzymatic Electrochemical Biosensors for Food Bioprocess Monitoring

In enzyme electrochemical biosensors, enzymes are used as recognition elements and immobilized on/inside the supporting substrate on the face of the transducer to maintain enzyme activity and enzyme stability due to the rapid reaction catalysis, the high-level substrate specificity and the comparatively long-term enzyme stability [21]. Binding enzymes as receptors can be easily adapted to monitor food quality and bioprocesses, and have a wide range of applications, especially in bioprocess analyses that require precise control and monitoring of substrate and product concentrations.

So far, there are three main systems of enzymes used for receptors, i.e., single-enzyme system, multi-enzyme system, and nano-enzyme system, where nanoenzymes refer to a class of nano materials that harbor enzymatic properties. In this section, the main focus will be on the construction principles of enzyme electrochemical biosensors and the research progress of these three systems in the food field in recent years.

### 2.1. Principle of Enzyme Electrochemical Biosensor Construction

**Figure 1:**
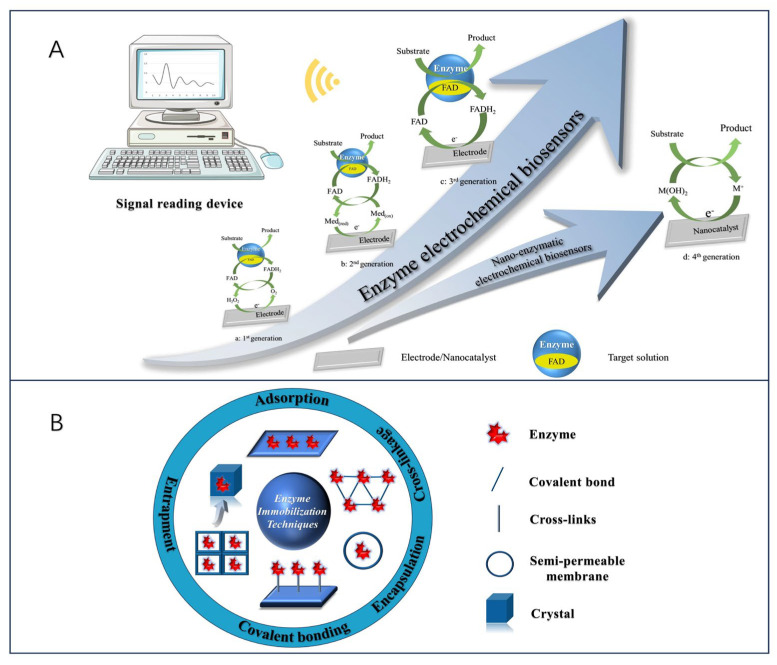
(**A**) Schematic of four generations of electrochemical biosensors. (a) 1st-generation biosensors use oxygen as an electron acceptor; (b) 2nd-generation biosensors use natural or artificial redox media as electron acceptors; (c) 3rd-generation biosensors use neither oxygen nor media, but enzymes can transfer electrons directly to the electrodes; (d) 4th-generation biosensors use nanomaterials to mimic various enzyme activity. (**B**) Schematic of the main methods for enzyme immobilization.

The development of enzyme electrochemical biosensors can be categorized into four generations (Figure 1A). The first generation of enzyme electrochemical biosensors was based on the measurement of analyte sample concentration based on the generation of H_2_O_2_ or by reducing the concentration of oxygen (O_2_) as a natural cofactor [22]. In this, enzymes use O_2_ as an electron acceptor and participate in the production of products (e.g., gluconic acid) [23]. In the initial stage, the flavin adenine dinucleotide (FAD) of the enzyme redox center acts as a catalyst to play the role of the initial electron acceptor, which is reduced to FADH_2_ in the analyte, and the reoxidation of FADH_2_ with free oxygen produces the oxidized form of the enzyme FAD. Typically, the analyte concentration corresponds to the electrochemical oxidation product, H_2_O_2_, or the electrochemical reduction product, O_2_, at the working electrode [24], and the transferred electrons are detected and collected by the working electrodes so that an analyte molecule’s number of atoms is proportional to the flow of electrons. However, this sensor is overly dependent on dissolved oxygen for electron shuttling, which may lead to fluctuations in oxygen tension due to the limited solubility of oxygen in the liquid to be measured, and thus properties such as hypoxia narrowing the linear range of the sensor can occur [25]. In addition, co-existing electroactive substances, such as acetaminophen (AP), ascorbic acid (AA), or uric acid (UA), may interfere with sensor use due to the high potentials required for the detection of H_2_O_2_ products [26]. To eliminate the dependence on oxygen, second-generation biosensors use natural or synthetic redox mediators to help electron movement between the enzyme and the underlying electrode, such as ferrocene and its derivatives [27], toluidine blue [28], and Prussian blue [29], which first react with the enzyme active site and then react with the electrode surface, thereby transferring electrons to generate a current signal proportional to the detected analyte concentration. In this process, the electroactive medium acts as a mediator to enable the biosensor to undergo mediated electron transfer (MET), also known as MET-type biosensors. In addition, the inclusion of an electroactive dielectric enables the second-generation biosensor to operate at low voltages and also avoids interference from coexisting electroactive substances. Although the second-generation biosensor is oxygen-independent, it is still subject to leaching and interference from the medium due to redox medium selectivity [30].

In contrast, the response of third-generation electrochemical biosensors occurs without the need for a medium. It solely depends on the interaction between the analyte and the bioreceptor, achieved by attaching the FAD-active redox center of the enzyme to the working electrode through nanomaterials either covalently or electrochemically. This arrangement enables direct electron transfer (DET), effectively eliminating any influence from the redox medium. In addition, free electron transfer was exhibited between the oxidoreductase and signaling sensor components, where the oxidoreductase acts as an electrocatalyst to facilitate the electron transfer between the electrode and the substrate molecules with excellent selectivity and sensitivity [11,31,32]. Nevertheless, there are some limitations to this sensor. Specifically, direct electron transfer between the enzyme’s deeply embedded FAD-active redox center and the working electrode is enhanced due to the leaching effect of the enzyme and the excellent conductivity of the nanomaterials. Therefore, a research priority to advance sensor technology lies in identifying suitable nanomaterials for electrode modification. Finally, the fourth generation of enzymatic electrochemical biosensors, also known as (nano-enzymatic) non-enzymatic biosensors. In this mechanism, atoms in the nanomaterial act as electrocatalysts to achieve direct electron transfer during chemical reactions [33]. Thus, by electro oxidizing the substrate, the nanomaterial substrate reacts to produce products, a process that shows great electrocatalytic efficacy [34]. However, as far as the nano-enzyme mimics found so far are concerned, they have limited substrate selectivity.

On the other hand, enzymes have properties as immobilized sensitive biological components that recognize the analyte and are usually immobilized on the surface of an electrode to detect the analyte. However, their inherently fragile nature can lead to early denaturation and short life, but this can be avoided by different methods of enzyme immobilization [35,36]. The five most common methods of enzyme immobilization are adsorption, covalent bonding, cross-linkage, entrapment, and electrochemical polymerization, as shown in Figure 1B.

One of the simplest methods is a reversible weak non-specific force adsorption process through non-covalent bonds such as hydrogen bonds, hydrophobic interactions, van der Waals forces, or other ionic bonds [37,38]. As such bindings are weak, the basic structure and activity of the enzyme is not altered and the enzyme is immobilized in a random orientation on the surface of the nanomaterial-modified electrode. Covalent bonding immobilization of receptors is one of the most commonly used methods of irreversible enzyme immobilization. Covalent binding immobilization of enzymes involves the formation of covalent bonds between one or more functional groups of the enzyme to share electron pairs [39], and the direction of enzyme binding can be controlled by chemical binding; in contrast to adsorption, the enzyme is firmly linked to the nanomaterial-modified electrode, ensuring long stability of the immobilized bioreceptor. Cross-linking is another irreversible method of enzyme immobilization that does not require support to prevent enzyme loss into the substrate solution and is carried out by the formation of intermolecular cross-links between enzyme molecules by bifunctional or multifunctional reagents such as aldol condensation of glutaraldehyde, ensuring a strong chemical bond [38]. Embedding is defined as an irreversible method of enzyme immobilization and is divided into two main types: entrapment and encapsulation [40], which can be encapsulated inside a carrier or fiber by polymeric membranes, gels, or nano-lattice materials such as metal-organic frameworks [41], allowing substrates and products to pass through but leaving the enzyme behind, ensuring the integrity of its properties. Another interesting method of immobilizing enzymes is the generation of polymers on the electrode surface by methods of electrochemical polymerization, in which the enzyme is immobilized in a 3D matrix (electropolymerized membrane, amphiphilic network, polysaccharide, etc.); this approach makes it possible to immobilize the enzyme, the medium, and the additives all together on the same detection layer, without the need to modify the biomolecule and ensuring that the immobilized enzyme is well protected during the immobilization operation. Moreover, the theory of immobilized biomolecular systems (Reaction at an Interface, Range of Forces Affecting Adsorbed Biospecies) is considered in Smutok and Katz’s article, and enzyme immobilization methods as well as signaling modalities are discussed in detail, which provides strong support for the research in this paper [42].

### 2.2. Electrochemical Biosensors for Single-Enzyme Systems

Electrochemical biosensors for single-enzyme systems have an important role in the monitoring of food bioprocess monitoring. During fermentation, it is possible to determine the speed of the fermentation process and the appropriate fermentation endpoints based on the magnitude of the concentration of the analyte, to obtain industrial products with good flavor and nutritional value. Due to their very stable catalysis of the oxidation-reduction reaction, oxidoreductases and peroxidases are the most widely reported enzymes in electrochemical biosensors. This chapter will concentrate on the research progress on electrochemical biosensors for enzymes such as glucose oxidase and lactate dehydrogenase in the monitoring of food bioprocesses.

#### 2.2.1. Glucose Oxidase

**Figure 2:**
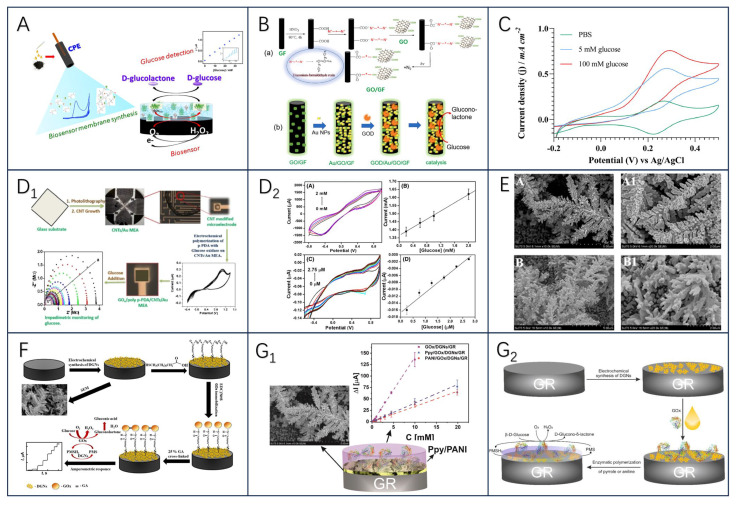
(**A**) Schematic diagram of poly−FA−GOx−BSA biosensing membrane synthesis, reprinted with permission [45], with permission of MDPI publications; (**B**) schematic illustration of the preparation of GO−modified carbon fibers by DR (a), AuNPs and GOD (b), reprinted with permission 51], with permission of Elsevier publications; (**C**) cyclic voltammograms were recorded at 1 mV s^−1^ for the optimized enzyme electrode. The enzyme electrode consisted of CNT−GOx (150 μg), Os(py)PVI (95 μg), and PEGDGE (34.2 μg), adopted from ref [48] with permission from the Elsevier publishers. Schematic construction of GO(x)/poly (p−PDA)/CNTs/Au MEA sensor and electrochemical response (**D_1_**); The CV plots of A GOx/poly (p−PDA)/CNTs/Si and C GOx/poly (p−PDA)/CNTs/Au MEA with different glucose additions at 20 mV/s in PBS at pH 6.5 are shown in (**D_2_**), and the corresponding calibration plots are shown in (**B**,**D_1_**,**D_2_**), reprinted with permission [52], with permission of SPRINGER LINK publications; (**E**) FE-SEM images of DGNs obtained after electrodeposition by CPA method (A, A1) and DPV method (B, B1), reprinted with permission [53], with permission of MDPI publications; (**F**) schematic of biosensor constructed based on GA−GOx−SAM/DGNs/GR electrodes, reprinted with permission [54], with permission of MDPI publications; the left panel shows the overall schematic(**G_1_**), and the right panel shows the schematic of the GR electrode modified with DGNs, followed by immobilization of GOx and enzymatic formation of a polymer (PANI or Ppy) layer for electrochemical glucose determination(**G_2_**), reprinted with permission [55], with permission of MDPI publications.

GOx is a glycoprotein with autophosphorylated proteins with reliable stability and substrate specificity. Glucose is an important hydrolysis product of food, and its concentration magnitude can be used as a key indicator for quality and process control. Electrochemical biosensors constructed based on GOx as a bioreceptor play an important role in food quality monitoring, especially in the control of biofermentation processes. The working principle is that the enzymatic oxidation of glucose produces H_2_O_2_, and then H_2_O_2_ and FAD, and FADH_2_ undergo an electrochemical reaction to be oxidized to produce O_2_, and when an operating current is supplied, the current generated in the sensing element is proportional to the amount of glucose present [43], which enables the detection of glucose concentration. Considering Clark’s study, many glucose-sensing electrodes were initially developed under the consideration of being based on enzyme membranes, which exhibit advantages such as high selectivity and high linearity [44]. For example, Valdés-Ramírez and Galicia synthesized biosensing membranes of ferulic acid (FA) and glucose oxidase on carbon paste electrodes via an electropolymerization reaction in aqueous media at neutral pH [45]. The results show that the novel poly ferulic acid membrane synthesized by electropolymerization is a promising method for immobilization of glucose oxidase and the developed glucose biosensor exhibits a wider linear glucose response compared with other polymer-based glucose biosensors (Figure 2A). The feasibility of synthesizing polyFA membranes in aqueous media with acidic, alkaline, and neutral pH was also demonstrated, increasing the potential of polymer membranes for the development of biosensing membranes. Furthermore, for the design of glucose biosensors, substances such as p-coumaric acid (p-CA) and vanadium dioxide (VO_2_) can be used [46,47].

In the last few years, to avoid the influence of complex environments on the final detection results, the sensors have mainly immobilized GOx on nanomaterial-modified electrodes, which are modified using nanomaterials or polymers to facilitate the electron transfer process and to reduce electrochemical interferences and the intervention of exogenous substances [48,49]. Another new idea is immobilization strategies using physical barriers (Nafion layer, etc.) to improve the instability of the enzyme layer [50]. As a recent example, Han et al. [51] demonstrated a novel approach to fabricating excellent electrochemical glucose biosensors using covalent bonding and self-assembly on graphite fiber (GF) surface (Figure 2B). Graphite fiber electrode (GFE) was modified using graphene oxide (GO)/gold nanoparticles (AuNP); GO and AuNP were interconnected along the GFE to form an efficient charge transfer channel, which, in addition to increasing the surface area, had the catalytic activity to prevent inactivation of the enzyme on the GFE surface. Moreover, the sensor’s detections were linear with glucose concentration, with a detection limit of 1.2 μM and excellent selectivity for dopamine (DA), UA, AA, and other interferents molecules (fructose, lactose, and galactose). Notably, the covalent bonding of GO with GF enhanced the contact between the electrode and the enzyme redox center and reduced the spacing between the electrode and the enzyme redox center.

Additionally, the stability and specificity of the enzyme were improved by using carbon nanotubes in glucose biosensors. Using covalently bonded nanoconjugates of GOx and MWCNTs (CNT-GOx) to improve the dispersion of the nanocarriers (Figure 2C), an electrochemical glucose biosensor based on osmium redox polymers cross-linked with GOx and GOx grafted on multi-walled carbon nanotubes (MWCNTs) has been reported to aid in the fabrication of enzyme electrodes and enhance enzyme activity [48]. After 50 h of continuous use, the stability was only 12 percent, but the Nafion protective layer increased the stability to between 72 and 75 percent, suggesting that engineering the relationship between the enzyme and the nano-support enhances the enzyme activity, thereby increasing the electrical density and enabling significantly lower amounts of active ingredients to be used. In a study by Singh et al. [52], by immobilizing GOx/poly(p-PDA)/CNTs/Au MEA) in a poly(p-phenylenediamine) matrix (Figure 2D_1_,D_2_) and by modifying CNTs/Au MEA, the selectivity of the resulting MEA for the detection of glucose was realized, whereby glucose could be separately measured for 64 samples with good reproducibility and immunity to interference, and the usability of the sensor was successfully verified by high-performance liquid chromatography (HPLC).

In another study, Ramanaviciene et al. [53] demonstrated the optimal scheme for the one-step electrochemical synthesis of dendritic gold nanostructures (DGNs) on graphite rod (GR) electrodes using three electrochemical methods, including constant potential amperometry (CPA), pulsed amperometry (PA), and differential pulse voltammetry (DPV), and the formed gold nanostructures (including DGNs) were characterized by field emission scanning electron microscopy (Figure 2E). The optimal HAuCl_4_ concentration (6.0 mmol L^−1^), DGNs synthesis time (400 s), electrodeposition potential (−0.4 V), and optimal electrochemical method (CPA) were determined; the sensors obtained by adsorption of GOx on the surface of DGNs and covalent crosslinking with glutaraldehyde (GA) vapor had linear ranges of up to 9.97 mmol L^−1^ (with dynamic ranges up to 49 mmol L^−1^), which has been successfully used for highly accurate glucose determination in real samples.

To further investigate the effect of DGNs as carrier nanomaterials on glucose biosensors, the team further analyzed the performance of GOx-immobilized sensors on DGNs as well as the development of enzyme biosensors with conductive polymer-modified DGNs in real-life samples [54,55]. The first results showed that covalently immobilized multilayer GOx on gold nanostructures is a very promising direction to improve the analytical parameters of biosensors; after covalently immobilizing GOx on a 11-mercaptoundecanoic acid self-assembled monolayer (SAM), the application of GA crosslinked GOx significantly improved the sensitivity and stability of the biosensor as well as the reproducibility of the current response after multiple glucose detection (Figure 2F). It is worth noting that DGNs are very fragile and may be damaged or detached from the surface along with the enzyme under inappropriate experimental conditions. The second study, on the other hand, demonstrated the significant advantages of the glucose biosensor designed with Ppy/GOx/DGNs/GR electrodes over PANI/GOx/DGNs/GR and successfully applied the constructed biosensor for the glucose concentration determination in authentic samples (Figure 2G_1_,G_2_). In conclusion, the immobilization of GOx on DGNs is of great importance for the further evaluation of glucose biosensors.

#### 2.2.2. Lactate Oxidase and Lactate Dehydrogenase

Lactic acid is the end product of sugar metabolism; L-lactic acid and D-lactic acid are the two isomers of lactic acid. L-lactic acid is a by-product of cellular metabolism indicating the transition from aerobic to anaerobic state, i.e., anaerobic metabolism produces L-lactic acid through the action of lactate dehydrogenase (LDH) as the end product of glycolysis [56], and its food products related to the fermentation and dairy industry sector is widely used [57,58]. In food quality testing and fermentation processes, electrochemical lactate sensors have been intensively investigated due to their low cost, simplicity, on-site detection, rapid response, portability, and minimal or no sample pretreatment [59]. Through these sensors, lactate oxidase (LOx) and lactate dehydrogenase (LDH) have been widely exploited, with a focus on electrochemical biosensors constructed with nanomaterial-modified electrodes, and a variety of nanoparticles including metals, metal oxides, mixed metal oxides, polymers, and composites have been investigated for L-lactic acid biosensing, with great advantages in terms of stability, selectivity, and improved sensitivity [60].

**Figure 3:**
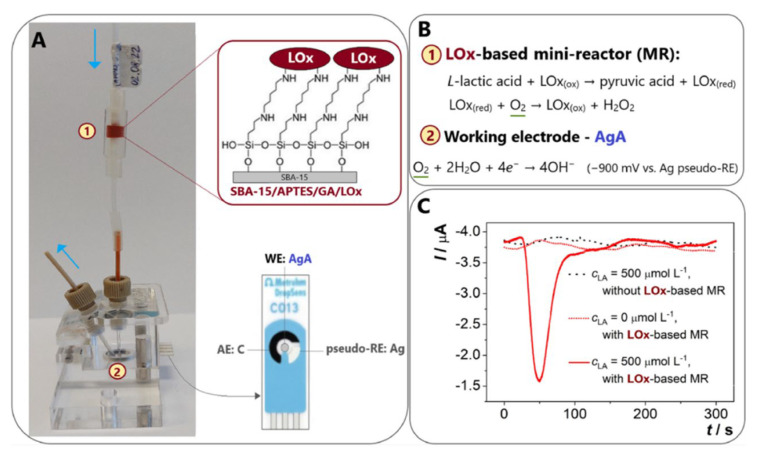
(**A**) Schematic diagram of the flow lactate biosensor. (**B**) Lactic acid detection schematic. (**C**) Flow type lactate sensor various types of amperage chart, reprinted with permission [65], with permission of Elsevier publications.

More recently, Narayanan and Slaughter prepared AuNP-cysteamine-LDH biosensing electrodes with good selectivity for lactic acid, and the electrodes obtained after coating with a Nafion layer remained stable for up to 18 days [61]. Istrate et al. [62] constructed a GA-LDH/AuNPs-ERGO-PAH/SPE disposable biosensor modified by a ternary composite of gold nanoparticles, electrochemically reduced graphene oxide, and poly (allylamine) hydrochloride on the surface of a carbon screen-printed electrode and crosslinked the immobilized enzyme with GA. The enzyme activity stability of LDH based on this construct was used for up to seven weeks. However, the use of LDH as a biologically active receptor means that the detection scheme is more complex compared to LOx-based biosensors, as the amperometric biosensing approach using LDH results in a complex biosensor structure due to the need for NAD^+^ as a mediator for shuttling electrons between the enzyme and the sensor [63]. Furthermore, the presence of additional compounds that are prone to oxidation (such as AA and UA) hinders the achievement of the necessary level of detection for NADH oxidation. This interference results in a heightened level of reversibility in the reaction involving lactic acid and pyruvic acid, ultimately impacting the sensitivity of the sensor and potentially causing blockages. Consequently, due to the aforementioned constraints experienced by LDH, there has been a greater focus on LOx, primarily due to the straightforward nature of its reaction. LOx plays a key role in the oxidation of lactic acid and its main function is to catalyze the conversion of lactic acid to pyruvic acid. In addition, the inactive state of LOx (red) is reduced in the presence of dissolved oxygen and this reduced form is subsequently oxidized to its active state, LOx (ox). The detection of Lox (ox) is achieved by electrochemically monitoring the generated hydrogen peroxide at highly positive potentials [64]. For example, Tvorynska et al. [65] developed a novel biosensing system for electrochemical flow injection analysis (FIA) that incorporates an easily replaceable LOx-based bioreactor biometric section (Figure 3). The microreactor consists of a mesoporous silica powder, SBA-15, coated with covalently immobilized LOx. Immobilization is achieved through the use of (3-aminopropyl) triethoxysilane (APTES) and GA. This immobilized LOx is referred to as SBA-15/APTES/GA/LOx. It is worth noting that the system is attached in front of an amalgam screen-printed electrode (AgA-SPE) that acts as a sensor. Oxygen consumption was monitored amperometrically by four-electron reduction with the Ag pseudo-reference electrode at a voltage of −900 mV, thus avoiding interference from common interfering compounds. The spatial separation strategy of the biorecognition and detection sections allows the immobilization of large amounts of enzyme (one microreactor contains ~270 μg LOx), thereby ensuring excellent operational and storage stability. The sensor greatly improves, simplifies, and saves the monitoring of lactic acid in biological samples for laboratory analysis and foods and wines for fermentation control and is now successfully used for the quantitative detection of lactic acid in saliva, wine, and dairy products. In another study, Ozoglu et al. [66] presented the design of an enzyme-based amperometric lactate biosensor with a linear range of 50–350 μM, a detection limit of 31 μM, and a sensitivity of 0.04 μA μM^−1^ cm^−2^ for the detection of lactate produced by six different, morphologically defined putative LAB. This study demonstrates that improving the interface of biosensors using a modification of composites or immobilization of mediators and enzymes on a catalyst layer is useful for designing interference-free measurement systems, especially for the detection of bacterial metabolites.

#### 2.2.3. Other Enzymes for the Development of Electrochemical Biosensors

**Figure 4:**
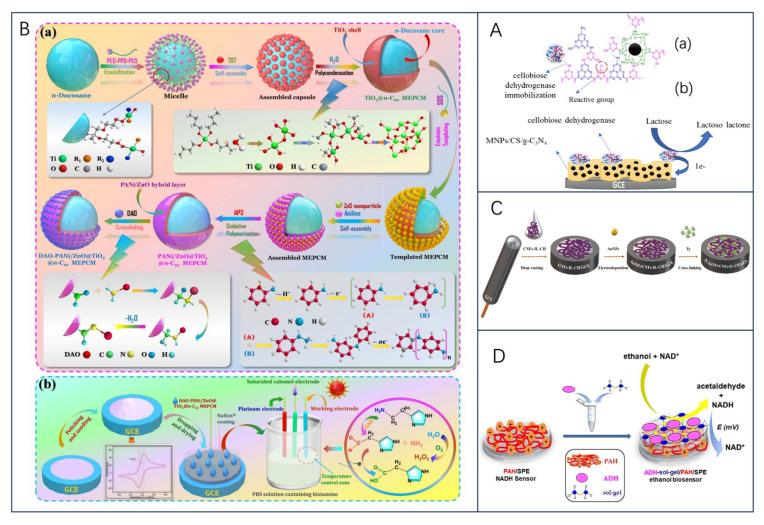
(**A**) Schemes of (a) functional groups on MNPs/CS/CDH and immobilization of CDH on MNPs/CS and (b) MNPs/CS/CDH/GCE and the electron transfer mechanism during lactose oxidation via CDH, reprinted with permission [68], with permission of Elsevier publications; (**B**) (a) preparation route and reaction mechanism of DAO-PANi/ZnO@TiO2@n-C22 MEPCM. (b) Schematic of the fabrication strategy and biosensing mechanism of DAO-PANi/ZnO@TiO2@n-C22 MEPCM-modified GCEs, reprinted with permission [73], with permission of Elsevier publications; (**C**) schematic illustration of the construction procedure of Ty/AuNPs/CNFs-IL-CH/GCE biosensor, reprinted with permission [74], with permission of Elsevier publications; (**D**) schematic illustration of the construction procedure of Ty/AuNPs/CNFs-IL-CH/GCE biosensor, reprinted with permission [63], with permission of MDPI publications.

Lactose, the major disaccharide in milk and dairy products, is formed by the β-1,4-glycosidic bond between galactose and glucose, and as a carbohydrate substitute, the sensitive detection of lactose content in food is an important factor in human health management [67,68]. It was found that carbon nanotubes interact well with lactase (LAC), and the biosensor obtained by immobilizing LAC with CNT had a sensitivity of up to 5.67 μA cm^−2^ mmol^−1^ L, with a limit of detection of about 100 × 10^−6^ mol L^−1^; and the stability of the system was improved with the introduction of CNT as, after about 12 h of use, the current signal did not change after about 12 h of use [69]. Building on this, the team further used only CNT as a substrate to immobilize LAC by adsorption without any polymer stabilization layer or external membrane for the rapid and sensitive detection of lactose in skimmed milk samples [70]. In this regard, Villalonga et al. [71] argued that the variations in the anodic and cathodic peaks in the article could be due to metal residues in the CNT, as well as to the influence of other enzymes or material components present in the enzyme preparation. Therefore, the finding could not be attributed to the adsorption of the non-oxidoreductase enzymes on the surface of the electrode in the article. Moreover, the electrode is not just a single-enzyme-modified electrode, since the signals analyzed are induced simply by lactose hydrolysis mediated by beta-galactosidase. In another work, Bollella and Gorton found that cellobiose dehydrogenase (CDH) is selective for lactose and therefore can be used as an alternative for constructing lactose-catalyzed biosensors [72]. Recently, Nasiri et al. [68] developed magnetic chitosan-supported graphitic nitride (MNPs/CS/g-C_3_N_4_) metal-free nanocomposite electrochemical lactose sensors by applying MNPs/CS/g-C_3_N_4_/CDH as a modifier to GCE electrodes, which exhibited excellent electrochemical performance within a large linear range up to 0.9–100 mm and a response time as fast as 5 s (Figure 4A). The sensor has great promise for real sample analysis and has been successfully validated for the quantitative detection of lactose in dairy products.

Biogenic amines (BA) are organic nitrogenous compounds naturally formed by bacterial decarboxylation of the corresponding amino acids in food, as a result of bacterial contamination under poor handling and storage conditions, and are considered to be a quality indicator of food freshness or deterioration [75,76]. And include histamine (His), tyramine (Tyr), dopamine, cadaverine (Cad), and putrescine (Put), among others, and excessive intake can affect human health [77]. Histamine is one of the main factors affecting the freshness of aquatic products. Monoamine oxidase (MAO) and diamine oxidase (DAO) as biorecognition molecules are modified on the electrode, which can effectively convert information such as analyte concentration into electrochemical signals with excellent selectivity and specificity [78]. Recently, X. Tian et al. [73] designed a temperature-regulated biosensor to better monitor histamine levels in high-temperature foods. The sensor is based on DAO-immobilized phase-change microcapsules, which are constructed by encapsulating n-docosane (n-C22) in a TiO_2_ shell, with a PANi/ZnO conductive layer deposited on the surface, and DAO is immobilized on the surface of the microcapsules (DAO-PANi/ZnO@TiO_2_@n-C22 MEPCM). The n-C22 core acquires thermoregulation through a reversible phase transition at high temperatures and can change the ambient temperature around the working electrode to improve the biocatalytic activity of immobilized DAO (Figure 4B). Compared with the conventional histamine biosensor, the biosensor had a detection limit of 0.473 mu mol/L at high temperature and a high sensitivity of 28.57 mu A.mM(^−1^).cm(^−2^). In another study, Erden et al. [74] investigated a novel amperometric tyramine biosensor (Ty/AuNPs/CNFs-IL-CH/GCE) modified with a composite membrane of carbon nanofibers (CNFs), chitosan (CH), ionic liquid 1-butyl-3-methylimidazolium tetrafluoroborate (IL), and gold nanoparticles (AuNPs). The results showed that the Ty/AuNPs/CNFs-IL-CH/GCE biosensor was highly selective for tyramine in the presence of other biogenic amines (Figure 4C). To further delve into the development and trends of biogenic amines in food safety applications, researchers conducted a comprehensive review of meat products [79] and fermented foods [80,81]. It is believed that amino acid decarboxylase is a key factor in the production of BAs, that convenient, rapid, accurate, and environmentally friendly methods for the detection of biogenic amines are emerging, and that the combination of physical and biological methods is a promising approach for the control of BAs. Future research could also focus on the development of a platform combining multi-sensor arrays and pattern recognition techniques for the high-throughput detection of biogenic amines.

The expanding global alcohol market has led to research on biosensors for the determination of ethanol content in alcoholic beverages. Here, Prasanna Kumar et al. [82] immobilized alcohol oxidase on carboxylated graphene/poly(diallyldimethyl ammonium chloride)-modified graphite electrodes and constructed a responsive biosensor system. In a recent study by O.-M. Istrate et al. [63], a screen-printed electrode was modified to detect ethanol in commercial beverages. The researchers used a sol-gel matrix to immobilize ethanol dehydrogenase on the sensor surface and applied a layer of poly(allylamine hydrochloride) to enhance the accumulation of NADH (Figure 4D). This modification led to an increase in the oxidation current of NADH, allowing for the accurate detection of ethanol. The sensor exhibited a sensitivity of 13.45 ± 0.67 μA/mM·cm^2^ and a detection limit of 20 μM, making it highly suitable for determining ethanol content in alcoholic beverages and foods.

In addition, the detection of pathogenic bacteria in food can be achieved using enzyme-based biosensors [83,84]. Xanthine oxidase-based biosensors can be used to detect levels of hypoxanthine and xanthine, which are markers of spoilage in meat [14], and hypoxanthine-sensitive electrochemical biosensors can detect fish freshness [85]. Nitrate reductase (NaR) or microorganisms containing NaR can be used to detect nitrate. Engineered L-glutamate oxidase can be used for monitoring glutamate during microbial fermentations [86], and Laccase can be used to improve food quality, determine phenols in tea and wine [87,88], etc.

### 2.3. Electrochemical Biosensors for Multi-Enzyme Systems

**Figure 5:**
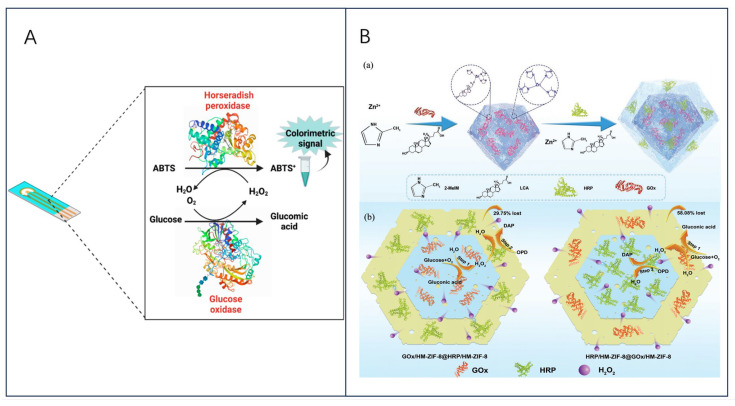
(**A**) Schematic representation of a bi-enzymatic biosensor, adopted from ref [92], with permission of MDPI publications; (**B**) (a) synthesis diagram of GOx/HM-ZIF-8@HRP/HM-ZIF-8, (b) schematical process of the cascade reaction in GOx/HM-ZIF-8@HRP/HM-ZIF-8 and HRP/HM-ZIF-8@GOx/HM-ZIF-8, reprinted with permission [93], with permission of Wiley Online Library publications.

To conveniently detect the products of one or more enzymatic reactions, avoid the inability of single enzymes to be catalyzed in electrochemical reactions, and effectively prevent the inhibitory effect of enzymes, researchers usually add multiple enzymes to the biorecognition elements of biosensors to form a multi-enzymatic system, which improves the performance of biosensors [11]. Kucherenko et al. [12] in their article reviewed the advantages and limitations of the development of multi-enzyme biosensors, gave suggestions on the rationality of novel multi-enzyme biosensors, and based on different enzyme-associated reaction principles can be categorized into biosensors based on enzyme cascade, cyclic enzyme-promoted, enzyme-competitive substrate, and enzyme-independent reactions. Among them, the biosensor based on enzyme cascade reaction is the most common type of multi-enzyme biosensor, which consists of several consecutive biocatalytic steps, i.e., the first enzyme converts substance A to the substance unstable intermediate B, the second enzyme converts substance B to C, and so on, to form the final stable electrochemically active product for detection (Figure 5A, which has the advantages of eliminating the need for intermittent product separation, saving cost and reagents, high reversibility and low inhibition, etc., and is widely used in food processing [89], disease treatment, and industrial production [90].

Currently, the longest enzyme cascade reaction in the biosensor consists of five enzymes including glycerol kinase/creatine kinase/creatinase/sarcosine oxidase/peroxidase [91], and has been successfully used to analyze glycerol in various white and red wine samples. However, although multi-stage material conversions using multiple enzymes are possible, in most instances the number of enzymes is limited to two because each additional enzyme has a different sensitivity to the substrate, a different method of enzyme immobilization, and a different storage time of the enzyme activity, and can cause an increase in sensor response time and higher manufacturing costs.

Multi-enzyme partitioning contributes to the optimization of the channels for substrate transport and promotes the controlled and tunable progression of the reactions in complex cascade biocatalysis. G. Wu et al. [93] proposed to partition GOx and HRP in a core-shell zeolite imidazolium framework (ZIF)-8@ZIF-8 nanostructure to construct a partitioned GOx/HRP dual-enzyme system based on core-shell ZIF@ZIF nanostructures (Figure 5B). Nucleation was induced using bionic mineralization and slow seeding to obtain a homogeneous shell wrapped around the core surface by epitaxial growth, whereas the dual enzyme system segregates in the shell and core. Meanwhile, the pore structure of ZIF was improved from a single microporous to a hierarchical microporous/mesoporous network to further improve the mass transfer efficiency, and the system can also covalently bind lithocholic acid (LCA) with divalent metal ions as a competitive ligand. Interestingly, the core-shell ZIF@ ZIF nanostructures proved to be versatile when adjusting the positions of the different ZIF types included or separated multi-enzymes, which provides a facile synthetic strategy for the development of efficient multi-enzyme biocatalysts.

Multi-enzyme cascade reactions can directly reduce biomanufacturing costs and increase productivity by eliminating the tedious isolation and purification steps required to remove reaction intermediates. However, the precursor condition used for multi-enzyme systems is that the enzymes to be found are all relatively similar in terms of sufficient activity and stability, and due to the inherent instability of the enzymes under non-physiological conditions, the low stability of the enzymes in practice, and the poor reusability, are all major challenges to be overcome [94]. Therefore, how to achieve the immobilization of a multi-enzyme system on an electrode platform in a way that ensures its activity and is stable is a question that needs to be investigated. Indeed, the number of publications related to enzyme immobilization has steadily increased over time, and co-immobilization of enzymes has also emerged as a fruitful approach for controlled multi-enzyme immobilization, where two or more enzymes can be confined in the same space and the immobilization sequences can be regulated to enable highly sensitive multi-enzyme systems for analyte detection [95]. In turn, immobilization of enzymes on carrier materials such as graphene, carbon nanotubes, metal–organic frameworks, and conductive polymers is one of the most effective methods to increase enzyme activity through substrate channeling and to improve enzyme stability and reusability [96]. For example, He et al. [94] immobilized GOx and horseradish peroxidase (HRP) in single-stranded DNA (ssDNA) ligated MOFs and graphene oxide (GO) dual carrier platforms ssDNA bridged the dual carriers and reduced enzyme leakage, and the dual carriers increased enzyme loading and accelerated enzyme efficiency. This enzyme immobilization strategy has a promising application in biocatalysis and diagnostics. This enzyme immobilization strategy has a broad application and practical value in the field of biocatalysis and diagnostics. Table 1 below shows a few examples of constructing electrochemical biosensors based on the immobilization of multiple enzymes.

**Table 1:** Cases of constructing electrochemical biosensors based on multienzyme immobilization.

Model Enzymes	Support Materials	Target Substance	Linear Reaction Range (μM)	Detection Limit (μM)	Application	References
HRP and GOx	G-IL/CNTs	glucose	0.004–5 mm	3.99 × 10^−7^ M	Determination in real samples	[97]
INV and GOx	INVWM-GOx-Au/CuNPs-MFC-IGT/AuSPE	sucrose	0.1 nM–10 μM	0.1 nM	Direct sucrose snalysis in sweetened beverages	[98]
GaOx and β-gal	P(Py-co-EDOT)-NaDBS	lactose	0.2–2.3 mM	1.4 × 10^−5^ M	Lactose Determination in milk samples	[99]
GOx and β-gal	Chitosan/Enzyme(s)/Chitosan/GA	lactose	5.83 × 10^−3^–1.65 × 10^−2^ M	1.38 mM	Determination of lactose in dairy products	[100]
GOx, β-gal, and mutarotase	PmPD	lactose	0.01–1.25 mM	0.005 mM	Determination of lactose in dairy products	[101]
HRP and LOx	Electrosynthesis PPy film	lactose	1 × 10^−6^–1 × 10^−4^ M	5.2 × 10^−7^ M	Monitor malolactic fermentation for winemaking	[102]
GK and GPO	GK/GPO/CHIT/TA/NPG/AuE	glycerol	0.1–5 mM	77.08 μM	Control of wine quality	[103]
GOx and LOx	Flexible electrode array with gold nanoparticles and Prussian blue	glucose lactose	60–1000 μM 5–20 mM	/	Medical diagnosis	[104]
GOx, CO, and HRP	MIPs/MWCNTs-IL/GCE	glucose cholesterol	1–18 pM 0.5–15 pM	0.81 pM 0.23 pM	Medical diagnosis	[105]
GA-bacteria and GDH-bacteria	MWNTs/GCE	Maltose Glucose	0.2–10 mM 0.1–2.0 mM	0.1 mM 0.04 mM	Monitoring of food production and fermentation processes	[106]
HRP and GOx	Polynoradrenalin/Polyaniline electrode	Glucose H_2_O_2_Cr(III) Cr(VI)	0.50 μM–0.42 mM 50–3.02 × 10^4^ 0.01~3.8 5.0 × 10^−4^~6.0 × 10^−3^	0.08 10 0.01 2.0 × 10^−4^	Determination in real samples	[107]

### 2.4. Electrochemical Biosensors for Nano-Enzymatic Systems

Even though enzymatic reactions are efficient and selective, they are still characterized by high cost, poor stability, difficulty in storage, and susceptibility of catalytic activity to the external environment, and there is an urgent need to find an effective way to solve these problems. In recent years, a new class of nanomaterials called nano enzymes (NZs) has emerged, which have catalytic activities that mimic those of enzymes and are expected to replace natural enzymes [108]. NZs are nanomaterials with unique physicochemical properties and mimic the properties of natural biological enzymes that perform the same kinetic behavior as natural enzymes and catalyze the conversion of substrates to oxidative coloration products [109,110], whose enzymatic activity is mainly caused by atoms on the surface of the nanoparticles and inside the core, and the nanomaterials, by coupling with biological molecules acting as oxidoreductases and used in the structure of NZs to provide high surface-to-volume ratios to enhance adsorption and advance electron transport, accelerate the analytical technique and show the advantages of being fast, sensitive, efficient, inexpensive, and having superior signals [13]. Thus, they are an effective alternative to address the weaknesses of natural enzymes [111,112]. Notably, the incorporation of various nanoparticles may alter the basic characteristics of NZs and may also make them multifunctional [113].

Since the discovery of the first nano enzymes (Fe_3_O_4_ NPs) in 2007 [114], materials such as metals, metal oxides, carbon nanomaterials, metal–organic frameworks, polymer-coated nanoparticles, and nanocomposites have been used for the synthesis of NZs [79,115], and some of these materials have been shown to have multiple enzymatic activities at once. For example, molybdenum disulfide (MoS_2_), simultaneously mimics the activities of superoxide dismutase, catalase, and peroxidase, whereas metal nanoparticles (NPs) are considered to have significant potential for analyte determination due to their abundant redox sites [116]; metal–organic frameworks (MOFs) are promising new materials due to their customizable pore sizes, functional groups, and biocompatibility, and are regarded as highly promising platforms in the study of enzyme–host material interactions [117].

Currently, NZs have been demonstrated to be used in the field of detecting glucose, phenols, hydrogen peroxide, pesticides, bacteria, cancer cells, and so on. However, since nanoenzymes are a novel technology, their official classification has not yet been determined. Huang et al. [118] suggested that NZs should be classified into two groups: oxidoreductases and hydrolases, and the family members of the oxidoreductase class have redox catalytic roles, which are usually used in biosensing applications, such as catalase, superoxide dismutase (SOD), oxidative enzymes, peroxidases, and nitrate reductase [119,120,121]. Similar to phosphatases, proteases, nucleases, esterases, and silicate lyases, hydrolases catalyze the hydrolysis process [115].

More recently, Smutok et al. [122] used a combination of two nanoenzymes with peroxidase activity and LOx in their work to construct an electrochemical lactate biosensor. Fragments of carbon microfibers (CFs) functionalized with hemin (H) and decorated with gold nanoparticles (AuNPs) or platinum microparticles (PtMPs) were used to synthesize the two nano enzymes. Among them, the constructed LOx-CF-H-PtMPs/GE nano-electrode showed good catalytic and operational characteristics in real sample detection. Q.-Y. Yang, Wan, et al. [123] constructed a metal–organic framework nano-enzyme BiO-BDC-NH_2_ using three-dimensional globular bismuth formate oxide (BiOCOOH) as a precursor and template, which possesses intrinsic peroxidase mimetic activity and efficiently catalyzes the oxidation of colorless 3,3′,5,5′-tetramethylbenzidine to produce blue oxidation in the presence of an enzyme. This product can be used for the detection of unlabeled and trace/super trace Cr^6+^.

In addition, several review papers have been related to the sensing applications of NZs, such as the research progress on NZs in food quality and safety detection [112], the NZs in integrated instant diagnostic biosensor development [115], and a comprehensive review paper on the application of NZs (single-atom enzyme) in the electrochemical monitoring of food safety and human health [124].

In summary, it can be seen that in recent years, nano enzymes biosensors have been developed rapidly and in a wide variety; therefore, in this section, the cases of representative nano enzymes in electrochemical biosensors mainly used in the field of food applications, such as nano-materials with peroxidase activity and oxidase activity, are listed in Table 2: Selected studies on nano enzymes based in food analysis, which is hoped to promote and inspire the research of electrochemical biosensors based on nano enzymes.

As can be seen in Table 2, most of the nano enzymatic biosensing studies have focused on redox enzyme-based nanomaterials (e.g., oxidases and peroxidases), which is mainly because the enzyme catalytic efficiencies of peroxidase and oxidase-based nano enzymes are slightly higher than those of the natural enzymes. Furthermore, despite all the advantages of nano enzymes, their applications still lack substrate specificity and have application limitations that need to be solved; therefore, there is a need for continuous research on the natural active sites of enzymes and the construction of new integrated nano enzymes systems to mimic and improve specificity. Binding or synergistic mechanisms of enzymes and nano enzymes have been reported to be a promising option to address this issue, as their interactions can improve the selectivity and sensitivity of these systems [122,125,126,127,128]. For better applications in areas such as clinical diagnostics, food analysis, and environmental monitoring, future work should concentrate on learning about the mechanism of action between nanomaterials and enzymes, as well as on the fabrication of novel materials with more enzyme similar activities.

**Table 2:** Selected studies on nano-enzymes based on food analysis.

Enzyme Mimicked	Nanomaterials	Target Substance	Linear Range	Detection Limit	Application	References
Oxidase	His@AuNCs/RGO	Nitrites	2.5–5700 μM	0.5 μM	Detection of nitrite in sausage samples	[129]
Oxidase	FeMnzyme	AA	8 μM–56 μM	0.88 μM	Determination of AA in actual kiwi fruit	[130]
Oxidase	Dex-FeMnzyme	TAC	1 μM–30 μM	1.17 μM	Practical applications in fruit and vegetable foods	[131]
Oxidase	MnO_2_ NRs	Pb^2+^	0.8–2500 nM	0.54 nM	Detection in actual sample oils, wines, and spirits	[132]
Peroxidase	AuPd@UiO-67	Hg^2+^	1.0 nM–1.0 mM	0.16 nM	Actual measurements of tap water and lake water	[133]
Peroxidase	Au_2_Pt NPs	TAC	/	<0.2 μM	Determination of TAC in real samples (milk, green tea, and orange juice)	[134]
Peroxidase	S-rGO	H_2_O_2_ glucose	0.1–1 μM 1–100 μM	0.042 μM 0.38 μM	Determination of glucose in real samples	[135]
Peroxidase	AgNPs/MoS_2_-MF	Glucose	1–15 mM	1.0 mM	Detection of glucose concentration in real samples	[136]
Peroxidase	Fe_1−x_S	Glucose AA	200–700 μM 10–500 μM	37 μM 53 μM	Detection of glucose and AA in actual beverages	[137]
Peroxidase	FeCo NCs	Histamine	1–5000 nM	0.79 nM	Detection of histamine in actual crab samples	[138]
Peroxidase	MOF-919-NH_2_@γ-CD	α-amylase activity	0–200 U L^−1^	0.12 U L^−1^	Determination of alpha-amylase activity in real distillers yeast samples	[139]
Peroxidase	PBA-CP@MOF	VP	102–108 CFU mL^−1^ 10–108 CFU mL^−1^	30 CFU mL^−1^ 5 CFU mL^−1^	Detection of VP in actual shrimp samples	[140]

## 3. Nanomaterials for Enzyme Immobilization

Enzyme electrochemical biosensor performance is largely influenced by three factors: morphology, structure, and enzyme immobilization technique, whereby the enzyme immobilization technique has the greatest impact on sensor performance. Immobilization of enzymes is almost mandatory for most of their applications [141], and in addition to advances in structural bioengineering of enzymes, methods of immobilization can range from random to charge-driven enzyme targeting—for example, stabilization by modification of functional groups on the enzyme or electrode surface, physical adsorption, covalent cross-linking, entrapment, or by incorporation into the cubic phase [73]. Currently, there are comprehensive reviews of technical methods for the arrangement control and enzyme immobilization of oxidoreductases on planar electrodes that have been published [142,143]. In addition, nanomaterials have also been used to address enzyme immobilization and are emerging as a dominant trend in current biosensor research.

With further research, the interaction between enzymes and different types of nanomaterial-modified surfaces such as metals and their oxides, graphene-related materials, metal–organic frameworks, conductive polymers, carbon nanotubes, etc., has been considered as a new strategy for enzyme immobilization [144,145]. Nanomaterial-modified electrodes can improve the rate and stability of electron transfer for enzyme immobilization, increase the sensitive surface of the sensor to immobilize more enzyme molecules, and have a fast response time due to their high conductivity that facilitates the rapid transfer of electrons from the redox region of the enzyme to the sensor [32,141]. Immobilization of appropriate enzymes close to nanomaterial-modified electrode surfaces is very effective for ensuring stable and efficient enzyme chemical biosensors, and it is a hot research priority to solve the enzyme immobilization problem [146].

In summary, the use of nanomaterials to modify electrodes to improve the various properties of sensors has become one of the main trends in the field of biosensing technology today, and therefore this section will focus on the progress of research on biosensors based on several nanomodified electrodes in the field of food engineering.

### 3.1. Metal-Based Nanomaterials Modified Electrodes

Metal-based nanomaterials (metals and their metal oxide nanoparticles) can be modified on the electrode surface to provide more binding sites for enzyme immobilization; in addition, combining with other nanomaterials can be involved in the immobilization of enzymes and further improves the conductivity and stability of the material, which is widely used in the field of electrochemical biosensors [147,148]. Metal-based nanomaterials commonly used for modifying sensor electrodes include gold (Au), silver (Ag), platinum (Pt), and iron (Fe), etc.; among them, gold nanoparticles (AuNPs) have been widely studied and used due to their excellent properties such as high electrical conductivity, high biocompatibility, catalytic activity, chemical stability, and nanocomposite modifications.

Research is currently being carried out on the application of AuNPs to various materials to improve the electrode performance of the sensors by immobilizing the AuNPs to significantly increase the activity of the enzyme through the formation of strong thiol bonds between the cysteine residues of the enzyme and the AuNPs. For example, Cerrato-Alvarez et al. [149] immobilized tyrosinase crosslinked with glutaraldehyde on the surface of screen-printed electrodes modified with gold nanoparticles (Tyr-AuNPS-SPCEs), and the fabricated sensors obtained good analytical and kinetic performance. In a further study, Narayanan and Slaughter constructed an improved electrochemical lactate biosensor by immobilizing LDH on a flexible tungsten microfilament electrode using a self-assembled monolayer (SAM) of cysteamine-modified AuNPs [62]. The sensor electrode (AuNP-cysteamine-LDH) remains stable for up to 18 days, and the Nafion layer used effectively shields the sensor from electrochemically active substances, resulting in excellent sensor performance at a potential of +0.4V, a temperature of 35 °C, and pH 6.

In addition, silver nanoparticles (AgNPs) and platinum nanoparticles (PtNPs) are common. To detect the presence of sugars (β-galactosidase, glucose oxidase, and galactose oxidase) in milk in combination with silver nanomaterials combined with biosensors to improve the performance of the multisensor system, Salvo-Comino et al. [150] developed a voltammetric bioelectronic tongue (bioET) specifically designed for the analysis of milk. The results show that silver nanowires (AgNWs) provide a more efficient platform than silver nanoparticles (AgNPs) for the immobilization of biomolecules, with unique performance characteristics in terms of sensitivity and detection limits. In another study, Sadak synthesized rGO/AuNPs nanocomposites and drop-cast them on SPCE for the preparation of enzyme glucose biosensors using GA as a cross-linking reagent and 2,5-dihydroxy benzaldehyde (DHB) as a medium using a one-pot method [151]. The protein cross-linking method was used to immobilize GOx on the pretreated SPCE to improve its electrochemical performance. Moreover, non-enzymatic electrochemical sensors based on dendritic polymers encapsulated with platinum nanoclusters and carbon nanotubes (Pt-DENs/CNTs) modifications have been developed for the determination of extracellular hydrogen peroxide (H_2_O_2_) released by living cells [152]. Based on these findings, the combination of multiple nanomaterials has been found to have greater advantages in improving the stability and sensitivity of biosensors.

Notably, metal oxides are also frequently used as modified materials for modifying electrodes, such as zinc oxide (ZnO), which is considered an excellent material for the preparation of high-performance electrochemical biosensors due to its intrinsic wide bandgap (3.2 eV), good biocompatibility, and better adsorption and catalytic properties [153]. Another aspect, by comparing pristine ZnO with Co-, Fe-, and Co-Fe-doped ZnO mixtures for glucose sensing, Baruah et al. [154] found that the Co-Fe-doped ZnO sensor modified with GOx showed a two-fold increase in sensitivity over the pristine sensor (32.2 μA mM^−1^cm^−2^), a linear range of 0–4 mM, and a response time of 6.21 s, demonstrating the advantages of composite nanomaterials in the field of biosensing. In addition, commonly used metal oxide nanomaterials include iron oxide (Fe_3_O_4_), titanium oxide (TiO), cuprous oxide (Cu_2_O) [155], and molybdenum oxide (MoO). For example, to achieve high-sensitivity monitoring of ochratoxin A (OTA) in real samples (fruit juice, red wine, and serum), Y. Wang et al. [156] proposed an aptasensor based on gold nanoparticle-modified molybdenum oxide (AuNPs-MoO), hybridization chain reaction (HCR), and restriction nucleic acid endonuclease (Nb.BbvCI)-assisted helper DNA machine aptasensor. In this electrochemical platform, HCR and Nb.BbvCI-assisted DNA walkers were used to achieve signal amplification, which demonstrated excellent analytical performance in the range of 0.01–10000 pg mL^−1^, with detection limits as low as 3.3 fg mL^−1^. In another study, Hui et al. [157] designed a sandwich-type electrochemical sensor based on AgNPs@Ti_3_C_2_ nanocomposites to detect Staphylococcus aureus in milk, where the self-assembled aptamer acts as a signal probe immobilized on CuO/GR nanocomposites by π−π stacking. The bacterial recoveries monitored by this sensor ranged between 92.64% and 109.58%, providing a new approach to the detection of pathogenic bacteria in food bioprocess monitoring.

### 3.2. Graphene Nanomaterials Modified Electrodes

Graphene (GR) is a class of monolayers of carbon atoms based on a honeycomb lattice arrangement. As a new type of carbon nanomaterial, GR has a two-dimensional (2D) conjugated structure, excellent electrical conductivity, high specific surface area, and satisfactory biocompatibility [158]. Graphene oxide (GO) and reduced graphene oxide (RGO) are functionalized derivatives of GR, which are widely used due to their abundant oxygen-containing functional groups, good biocompatibility, and excellent electrochemical properties [159], whereas RGO is mainly synthesized by reducing GO through various chemical methods [160]. It was found that while the defects and functional groups of GO favored enzyme immobilization and gained high sensitivity detection properties at the expense of electron transfer ability, reduced GO balanced both [161]. One study investigated the effect of GO reduction on glucose detection, and they found that the surface functional groups of partially reduced GO favored GOD uptake, while highly reduced GO facilitated rapid electron transfer, suggesting that an increase in the number of oxygen functional groups leads to an increase in GOD uptake, which in turn improves the affinity and sensitivity of the biosensor. With further research, scientists have found that RGO has become the most effective transducer material for biosensor design due to its high surface area, abundant functional groups, ultra-high electron mobility, remarkable electrocatalytic properties, and good electrical conductivity [162,163,164].

**Figure 6:**
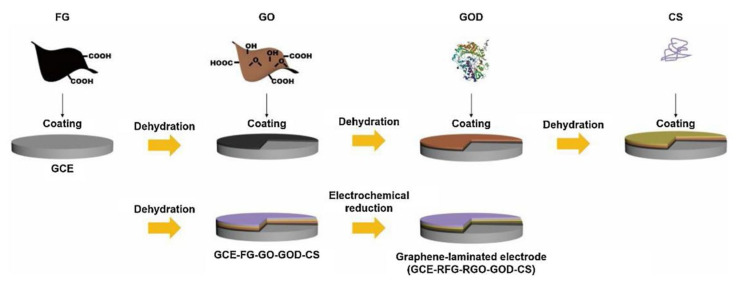
Schematic of the preparation of graphene laminated electrode (GCE-RFG-RGO-GOD-CS), reprinted with permission [166], with permission of Elsevier publications.

In addition, to obtain high GOD loading and highly sensitive biosensor detection properties, Fang et al. [165] prepared edge-modified multilayered graphene with high structural integrity, which demonstrated its great potential in preparing multifunctional nanofillers for high-performance composites; on this basis, Hao et al. [166] combined GO and edge-functionalized graphene (FG) layers combined onto a glassy carbon electrode to prepare graphene laminate electrodes (Figure 6). Due to the rich functional groups of GO, the high conductivity of FG, and the strong interactions between the components in the graphene-laminated electrode, the graphene-laminated electrode exhibited a faster electron transfer rate, a higher GOD loading of 3.80 × 10^−9^ mol cm^−2^, and a detection sensitivity as high as 46.71 μA mM^−1^ cm^−2^.

Due to its good electrochemical properties and biocompatibility, graphene is also frequently used for biosensing by making nanocomposites with other materials such as metal nanomaterials, metal–organic frameworks, Mxene, carbon nanotubes, quantum dots, and conductive polymers [167]. It has been found that GOx covalently immobilized on GR electrodes modified with DGNs is a very promising direction to improve the analytical parameters of biosensors [54,55]. In contrast, Popov et al. [168] attempted to use a GR electrode pre-modified with the conductive polymer polyaniline (PANI) and rGO, Nafion, and GOx dispersions as a working electrode for biosensors, and the developed glucose biosensor had wide linear range (0.5–50 mM), a low detection limit (0.089 mM), and good reproducibility.

On the other hand, metal–organic frameworks (MOFs)/graphene nanomaterials can be easily transformed into structurally complex materials (carbonaceous materials, metal carbides, etc.) due to their compositional and structure modifiability; moreover, the stable chemical interface between MOFs and GO/rGO is an effective way to improve the various properties of the sensor. However, other factors such as enzyme catalytic activity and reusability should also be concerned when designing biosensing platforms [169,170]. Indeed, biosensing platforms with high performance have been constructed by combining MOF/GO [171]. In conclusion, graphene-based nanocomposites are currently a promising option for the development of electrochemical biosensors.

### 3.3. Metal-Organic Framework Modified Electrodes

Metal–organic frameworks (MOFs) are porous crystalline materials consisting of metal ions or clusters bonded to organic linkers through coordination bonds [172]. Its high porosity, large surface area, tunable pore size, highly ordered pore structure, and good stability enable MOF to provide suitable sites for enzyme attachment and can be used as an effective platform for the construction of various chemical sensors and biosensors [173,174]. Currently, biosensors prepared with MOFs materials have been applied to various fields such as food safety and food quality control [175,176,177,178]. However, due to the poor conductivity and poor surface affinity of MOF, the performance of most MOF-based biosensing platforms so far has not reached the desired level. Currently, researchers are working on introducing nanomaterials with good conductivity into the MOF to modify the bioelectrode, which in turn improves the efficiency of electron transfer between the enzyme and the electrode. For instance, Xiao et al. [179] enhanced the biocatalytic effect of the substrate by in situ growth of ZIF-8 nanoparticles ZIF-8/GO composite on the GO surface, which enhanced the substrate biocatalytic effect with the enzyme by co-sedimentation under mild conditions and catalysis, and obtained a sensor with high sensitivity, reproducibility, and good stability. C. Chen et al. [180] combined hydrophilic multi-walled carbon nanotubes (HKUST-1-MWCNTs) with good electrical conductivity with copper-based MOFs and used a one-pot method to prepare a biosensing platform based on PDA-enzyme-HKUST-1-MWCNTs, which was carried out by the high porosity of HKUST-1 and the good adhesive property of PDA immobilization. The sensitivity of this sensor for glucose was 178 mu A mM(^−1^) cm(^−2^) over a wide linear range of 0.005–7.05 mM, and the detection limit was 0.12 mu M, with a corresponding RSD of 3.8%.

**Figure 7:**
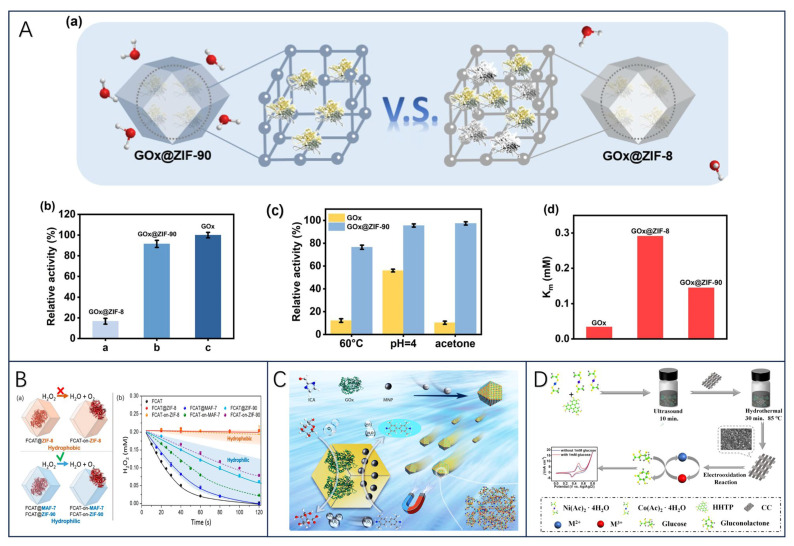
(**A**) (a) Schematic representation of the enzyme encapsulated in different frameworks; (b) relative activities of GOx, GOx@ZIF-8 and GOx@ZIF-90 under equivalent conditions; (c) stability changes of GOx and GOx@ZIF-90; and (d) comparison of the Km of GOx, GOx@ZIF-8, and GOx@ZIF-90; reprinted with permission [182], with permission of Elsevier publications. (**B**) (a) Schematic representations of the different FCAT/ZIF biocomposites formed by encapsulation of enzyme molecules via biomimetic mineralization or surface adsorption within/on hydrophobic (orange) or hydrophilic (blue) frameworks; (b) catalytic activity of FCAT and different FCAT/ZIF composites; reprinted with permission from [183], copyright 2019 American Chemical Society. (**C**) Schematics of synthesizing amorphous magnetic framework composites GOx/MNP@aZIF-90 and the catalytic activity Stues, reprinted with permission from [184], copyright 2022 American Chemical Society. (**D**) Schematic illustration of the synthetic process of the Ni/Co(HHTP)MOF/CC and their application in glucose determination, reprinted with permission [185], with permission of Elsevier publications.

In addition, because of the MOFs’ weak electrical conductivity and low surface affinity, X. Liu et al. [181] proposed a new strategy to address them: (i) the use of MOFs with their catalytic properties towards the substrate to enhance the synergistic catalytic effect of the combination of MOFs and immobilized enzyme; (ii) introducing hydrophilic carbon nanomaterials to prepare MOF/carbon nanocomposites to improve the electrical properties of the materials and the surface affinity of the enzyme-substrate to the hydrophilic nanocomposites. This strategy indirectly demonstrates that hydrophilic metal–organic skeletons can significantly enhance enzyme immobilization and protection, while promising the design of relevant MOF nanocomposites, which will be beneficial for the development of biosensing technologies. In another study, since zeolite imidazole framework-90 (ZIF-90) can modulate interfacial interactions to maintain the catalytic activity of the encapsulated enzyme, Ge et al. [182] designed a cascade catalytic reaction in which ZIF-90 encapsulated with GOx was combined with Pt NPs (GOx@ZIF-90-Pt NPs) for biosensing (Figure 7A). The results showed that the activity of GOx in GOx@ZIF-90 (90%) was 4.5 times higher than that of GOx in GOx@ZIF-8 (20%) when the catalytic activity of free enzyme was set at 100%. Meanwhile, GOx@ZIF-90 showed a 2.0-fold increase in substrate affinity over GOx@ZIF-8, promoting its potential application in biosensing.

Recently, W. Liang et al. [183] investigated the effect of hydrophilic and hydrophobic MOFs on the activity of encapsulated enzymes by finding that hydrophobic ZIF-8, on the other hand, provided inactive catalase and negligible urease protection, whereas the enzymes encapsulated in hydrophilic MAF-7 or ZIF-90 retained their enzymatic activity in the presence of high temperatures, protein hydrolyzing agents, and organic solvents (Figure 7B). It was demonstrated that hydrophilic MOFs provide considerable protection to enzymes loaded therein, whereas hydrophobic materials do not provide the same degree of protection. This study suggests that optimizing the hydrophobic/hydrophilic interaction between the enzyme and the encapsulation material is essential for efficient encapsulation and improved stability of the biomolecule, which is highly protective for the enzyme in acidic environments during fermentation.

In another study, Ji et al. [184] attempted to develop biocatalysts with multifunctional properties by combining nanoenzymes with natural enzymes to form a cascade reaction in response to enzyme instability and mass transfer barriers in sensor systems. Magnetic nanoparticles (MNP) and GOx were encapsulated in aZIF-90 (GOx/MNP@aZIF-90) by using amorphous ZIF-90 (aZIF-90) as the host material (Figure 7C). aZIF-90 generates mesopores and internal voids that effectively enhance the performance of the enzyme cascade reaction and provide confined protection against this reaction. The final results show that aZIF-90 exhibits almost four times the catalytic activity of the crystalline composite and has a residual activity higher than 80% after 9 days of storage. This is the first time that both GOx and MNP have been confined in aZIF-90 with mesopores, suggesting that amorphous metal–organic frameworks are promising in the development of enzymatic cascades.

In addition, MOF can also be utilized for the preparation of electrochemical enzyme-free glucose sensors, as demonstrated in Z. Xu et al. [185] where conductive Ni/Co bimetallic MOF [Ni/Co(HHTP)MOF/CC] was directly grown on carbon cloth via a simple hydrothermal method. Due to the synergistic catalytic effect of Ni and Co elements and good electrical conductivity, the bimetallic MOF and CC provided more catalytically active sites and larger specific surface area, and the prepared Ni/Co(HHTP)MOF/CC exhibited excellent electrocatalytic performance (Figure 7D) and was applied in real samples. The final results demonstrated that the sensing platform had a linear range of 0.3 mu M–2.312 mM with an LOD of 100 nM, a fast reaction time of 2 s, and a high sensitivity of 3250 mu A mM(^−1^) cm(^−2^).

### 3.4. Carbon Nanotube-Modified Electrodes

Carbon nanotubes (CNTs) are hexagonal sp2 hybridized carbon/graphite sheets rolled concentrically in a specific manner, dominated by single-walled carbon nanotubes (SWCNTs) and multi-walled carbon nanotubes (MWCNTs) depending on the number of graphene sheets rolled into the tube. Due to its inherent desirable properties such as high biocompatibility and high electrical conductivity, it is widely used in the field of biosensing [156,185,186].

Growing carbon nanotubes directly on the working electrode in situ is a strategy to take advantage of their electrochemical properties. Singh et al. [53] used carbon nanotubes grown in situ at low temperatures and imprinted a lithographically defined gold microelectrode array (CNTs/Au MEA) on a glass substrate for glucose detection. GOx was immobilized in a poly(p-phenylenediamine) matrix (GOx/poly(p-PDA)/CNTs/Au MEA), and CNTs/Au MEA electrode arrays were prepared to exhibit high conductivity and high enzyme loading due to the high surface area of the CNTs themselves and enzyme selectivity. The sensing platform shows good electrocatalytic properties and can individually detect glucose levels in 64 samples.

In addition, CNTs, as a special material, have been found to have a great capacity to be used in combination with enzymes [187]. H. Song et al. [188] constructed a hybridized system consisting of poly(vinylglycerol) swing-arm tethered NAD(+) and xylose dehydrogenase (XDH) with platinum nanoparticles (PtNPs@MWCNTs) deposited on carbon nanotubes for real-time sensing of xylose. The use of PtNPs@MWCNTs composites improved the sensitivity of the electrical response, significantly reduced the oxidation potential of NADH, and maintained 30% of the initial performance after 82 days, demonstrating its great potential for practical applications.

A study conducted by Maity et al. [189] involved immobilizing GOx on MWCNT/polyaniline/rGO/AuNPs/GCE to construct a glucose biosensor. The biosensing system achieved promising results, including 90.23% reproducibility (based on seven trials) and high stability of 96% (74.5% after 30 days of storage at −20 °C and 2 weeks of storage at −4 °C). In addition, the biosensor has a wide linear range of 1-10 mM, a low detection limit of 64 µM, and a high sensitivity of 246 µA cm(^−2^) mM(^−1^). Similarly, chitosan-based glucose biosensors were immobilized on polypyrrole (PPy)-Nafion (Nf)-functionalized MWCNTs to develop high-performance glucose biosensors [190]. The resulting nanohybrid composites provided a large surface area for GOx immobilization leading to high enzyme loading and hence improved sensitivity.

In summary, it has been shown that bio-nanocomposites prepared from CNTs with MEA, metal nanoparticles, metal–organic skeletons, and conductive polymers provide a biocompatible environment that can help increase the electrocatalytic activity of immobilized enzymes, enhance the electron transfer rate and improve properties such as high immunity to interference, longevity, reusability, and storage time [187].

### 3.5. Polymer Modified Electrodes

Conductive polymers, such as poly(aniline), poly(pyrrole), and poly(acetylene) are extensively employed in electrochemical biosensors to facilitate electron transfer between the enzyme and the electrode, as well as to enhance enzyme immobilization [191]. These polymers are particularly useful in oxidoreductase-based biosensors, where charge transfer is essential [192]. Compared to biosensors without polymers, biosensors incorporating conductive polymers exhibit heightened sensitivity due to the significant improvement in electron transfer between the enzyme active center and the electrode surface [20].

And one-step in situ electropolymerization of conducting polymers in the presence of monomers and enzymes has developed into an important and easy method for enzyme immobilization. A single-step procedure for the modification of graphite electrodes with polypyrrole (PPy), Prussian blue (PB), and GOx-based composite layers (PPy/PB/GOx) was investigated [193]. In addition, poly(3,4-ethylenedioxythiophene) (PEDOT), polydopamine, and silica are often used for one-step in situ enzymatic polymerization [194]. J. Li et al. [195] designed and constructed a GOx/AuNP/PEDOT(BSA)/Pt electrode platform for glucose sensing. The platform was determined to have a detection range of 0.416–50 mM by linear voltammetry, and the average value of sensitivity was about 3.124 mu A/mM/cm(^2^). Additionally, the electrode’s stability was demonstrated through uninterrupted glucose measurements spanning seven consecutive days, exhibiting a margin of error of approximately 5%. In the study conducted by Senel et al. [196], a groundbreaking film with exceptional conductivity was generated through the electrochemical polymerization of pyrrole (Py) along with thiophene-grafted chitosan (Th-Ch). The remarkable Ch-based conductive film was further enhanced by the incorporation of GOx, leading to a significant boost in sensitivity, surpassing the Py-Ch composite by approximately 40%. This novel composite film is promising in biosensor technology due to its biocompatibility, chemically and physically modifiable structure, and its conductivity.

**Figure 8:**
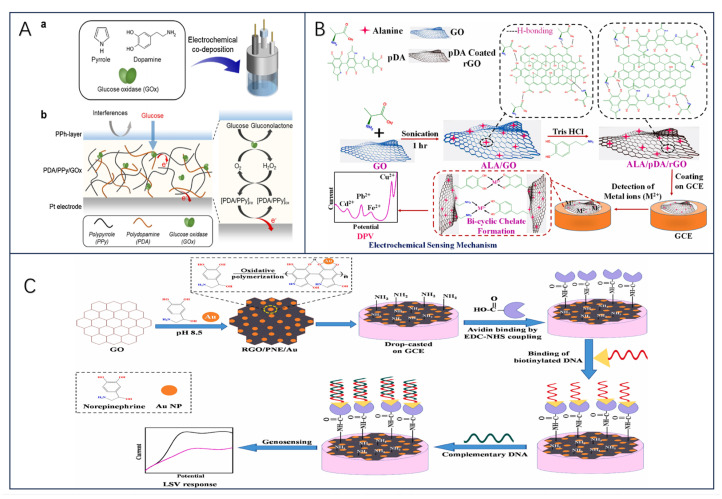
(**A**) (a) One-pot chronopotentiometric co-deposition of GOx and PDA/PPy on electrodes; (b) the sensing mechanism of the PDA/PPy/GOx amperometric glucose sensor, reprinted with permission [199], with permission of Elsevier publications. (**B**) Schematic representation for the fabrication of ALA/pDA/rGO ternary nanocomposite-based electrochemical sensor for the detection of HMIs, reprinted with permission [200], with permission of Elsevier publications. (**C**) Schematic showing the synthesis of RGO/PNE/Au nanocomposite for fabrication of DNA biosensor, reprinted with permission [164], with permission of Elsevier publications.

Besides conductive polymers, some biopolymers such as polydopamine (PDA) and polynephrine (PNE) have been widely reported and applied in the field of biosensors. PDA, as a biopolymer, has a wide range of functional groups that can be used for surface functionalization/nanocoating of materials through covalent bonding (acting as a cross-linking agent) or non-covalent bonding effects with the substrate, including metal coordination, π−π stacking, and hydrogen bonding, etc. [197,198]. A biosensor for glucose and lactate was developed through a one-step electrochemical coating process by M. Lee et al. [199] The GOx biosensor exhibited an impressive sensitivity of 22.15 A mM cm, a rapid response time of 5–6 s, a wide linear range of up to 5.0 mM, and a remarkable glucose detection limit of 138 μM (R = 0.995). Furthermore, the PDA/PPy/LOx biosensor exhibited enhanced lactate sensing capabilities in comparison to the PPy/LOx sensor (Figure 8A). This straightforward fabrication approach involving PDA/PPy and enzymes holds great promise in developing biosensors that are both highly sensitive and stable.

In another study, Patel et al. [200] prepared alanine-decorated polydopamine-coated reduced graphene oxide (ALA/pDA/rGO) nanocomposites, and the developed ALA/pDA/rGO was used for simultaneous electrochemical detection of Cd^2+^, Pb^2+^, Cu^2+^, and Fe^2+^ in solution (Figure 8B). Efficient detection of targets can also be realized in combination with a variety of nanomaterials [180,199,201]. Notably, inspired by the exponential growth of PDA research, scientists investigated the structure of PDA’s sister compound, polynorepinephrine (PNE), and found that PNE has greater coating uniformity and biocompatibility than PDA, which facilitates electron transfer between the enzyme’s active centers to the electrode [202].

Notably, the PNE chemical structure has one more -OH than PDA and contains abundant amino and hydroxyl groups with strong metal chelating and redox capabilities [203], allowing the material to serve as a multifunctional platform for surface functionalization, which has a great potential for application in the field of biosensors [204]. For example, Y. Liu, Nan, et al. [202] prepared Au electrodes modified by PNE, GOD, and AuNP (PNE/GOD/AuNPs@ PNE/Au), a sensor with excellent selectivity and stability. On the other hand, Bisht et al. [164] developed RGO/PNE/Au nanocomposite-based sensors for TB diagnostics and found that PNE-modified bioelectrodes have better DNA loading, sensitivity, and excellent electrochemical response; their findings further emphasize the importance of PNE-based biomimetic nanocoatings for the evolution towards the design of electrochemical biosensors for the significance of electrochemically active nanomaterials such as GR, RGO, MXene, etc. where functional groups are missing (Figure 8C).

## 4. Challenges and Future Trends of Enzyme Electrochemical Biosensors

### 4.1. Challenges

According to this article’s explanation about the sensors, it is evident that exploiting the distinct electrochemical characteristics of nanomaterials as modifications on electrode surfaces is an ingenious approach to enhancing the efficacy of electrochemical biosensors. This method facilitates the provision of additional electrocatalytic sites and immobilized sites for biomolecule binding. Nevertheless, despite numerous research endeavors focusing on biosensor development, the utilization and optimization of enzymatic-based electrochemical biosensors encounter various obstacles. These hurdles encompass:

(1) The major hindrances to the widespread usage of enzyme electrochemical biosensors are still the reusability and stability of these biosensors. Moreover, the complexity of food matrices, harsh environments, and their interference with biorecognition elements can significantly impact the reproducibility and selectivity of biosensors. Henceforth, scientists must prioritize the enhancement of sensor efficacy in forthcoming research endeavors. Specifically, rigorous investigation is necessary to address and resolve the issue of interferences encountered in authentic specimens, ensure the endurance of enzyme–chemical biosensors in adverse surroundings, and assess the impact of varying storage conditions on the biosensors’ lifespan [85].
(2) The addition of multiple enzymes to a biosensor in multi-enzyme systems can create complications during biosensor fabrication. Furthermore, it can impose substantial limitations on the characterization and application possibilities of the biosensor. This arises due to variations in the sensitivity to substrates, effectiveness in storage, and conditions required for enzyme immobilization among different enzymes. Hence, a critical consideration in designing a multi-enzyme biosensor is the meticulous selection of enzyme systems. This selection aims to prevent their sensitization to substances other than the target substance and ensure the requisite stability of the biosensor.
(3) Compared with natural enzymes, the catalytic activity of nano enzymes is still relatively low, and most nano enzymes are difficult to catalyze a specific substrate like biological enzymes. Therefore, despite all the advantages of nano enzymes, nano enzymes with high catalytic activity, excellent selectivity, and specificity for constructing nano enzymes-based biosensors still need to be further developed. In the future, integrating biological enzymes or nano enzymes into mesoporous nanomaterials to prepare integrated nano enzymes (INAzymes) or constructing a binding or synergistic mechanism between an enzyme and a nano-enzyme may be a promising strategy to obtain this type of problem [205].
(4) Achieving high homogeneity, reproducibility, and chemical stability in electrode materials is a challenging task that cannot be accomplished by simple synthesis alone. Obtaining these desirable properties requires continuous efforts to advance advanced synthetic methods and their application to the analysis of real samples. Therefore, future prospective studies could prioritize the assessment of the stability of biosensor electrode materials in complex environments. In addition, it would be beneficial to explore more reliable modification strategies to enhance compatibility between biorecognition molecules and electrodes, as well as other potential avenues of exploration.
(5) Enzyme orientation is an important influencing factor in the field of enzyme electrochemical biosensor construction, especially in terms of interfacial electron transfer. If the active sites of enzymes are used as binding sites to the electrode surface, then they cannot react with the target molecule and electron flow cannot be achieved. Therefore, before choosing the immobilization method, the enzyme can be controlled in the targeted distribution by focusing on the structural properties of the enzyme, the development of engineered enzymes with specific sites, as well as suitable surface modification techniques, or the use of (functionalized) nanoporous materials (noble metals, carbon nanomaterials, metal–organic frameworks, etc.) [18,150,206,207,208]. Additionally, enzyme orientation can also be performed by further in-depth studies of enzyme immobilization methods (e.g., random enzyme orientation due to physical adsorption, enzyme orientation resulting from the binding of functional groups in chemical cross-linking, and encapsulation of enzymes through the use of modified polymers, etc.) [18,40]. A recent study has shown that it is possible to regulate the orientation of the enzyme dipole moment by applying an external electric field (EF) to small molecules thereby enabling the correct orientation and deposition of biomolecules on surfaces [209].

While these factors present challenges to the commercialization of electrochemical biosensors, they have demonstrated exceptional capabilities in ensuring the analysis of food industry analytes as well as precise monitoring for bioprocess monitoring. Taken together in the full article, it is clear that the application of nanomaterials is expected to enhance the selectivity, sensitivity, storage stability, and other analytical properties of electrochemical biosensors. This enhancement is expected to make biosensors more resilient and expand their potential for practical applications.

### 4.2. Future Development Trends

Nowadays, with the development of science and technology, the requirements for biosensors that can achieve multi-functional rapid real-time monitoring are getting higher and higher, and integration and automation will also become one of the future development trends of biosensors. The integration and automation of smart devices in the food industry have the potential to greatly enhance the monitoring of food-related analytes. By integrating detection and analysis technologies, this approach can streamline and simplify the process of preparing biosensors. In addition, it has the potential to reduce the cost of sensors, making them more accessible for widespread use. Most importantly, these advancements are crucial for the development of real-time, online detection of target analytes during the monitoring of food bioprocesses [156]. For example, with the help of smart microelectromechanical systems (MEMS) and nanotechnology, biosensors can be miniaturized to the micron and nanometer scale and integrated into lab-on-a-chip devices for integrated and intelligent high-precision monitoring. Forouzanfar et al. [210] developed a carbon-microelectromechanical system (C-MEMS)-based highly sensitive electrochemical capacitive lactase sensor. The sensor showed good selectivity and high stability for lactate detection over a wide dynamic range of 0.1–5000 mu M with a detection limit of 1.45 mu M (signal-to-noise ratio = 3). In addition, it is often applied for rapid and efficient detection of product quality and safety [73].

In addition, self-powered biosensors have gained scientific interest; self-powered electrochemical biosensors utilize biofuel cells as a simultaneous power source and biosensor, which simplifies the biosensor system. The possibility of realizing self-powered biosensors for glucose detection was first demonstrated by Katz et al. [211] in 2001, and a comprehensive overview of enzyme-modified electrodes for biosensors and biofuel cells was provided by [212]. Recently, there have been advancements in developing cost-effective and user-friendly paper-based biosensors that are disposable [213,214,215]. For instance, an innovative study by Pagkali et al. [216] introduced electrochemical paper-based analytical devices (ePADs) with a fluidic setup. These ePADs were designed for creating enzyme-based biosensors by immobilizing GOx and utilizing potassium ferricyanide as a mediator within the designated test region. The fabrication of these biosensors incurred a manufacturing cost of less than EUR 0.05 each, making them highly affordable. By utilizing ePADs, the determination of glucose in food samples was successfully performed, exhibiting remarkable recoveries ranging from 94% to 106%.

## 5. Conclusions

In this review, an effort is made to compile the most recent developments in electrochemical biosensors for food analysis and bioprocess monitoring utilizing enzymes. Nevertheless, given the vast number of samples, it becomes impractical to offer a comprehensive survey of all sensors. Therefore, this review exclusively presents a chosen range of well-known enzymes employed in biosensors. Among enzyme-based biosensors, those constructed as single-enzyme systems, multi-enzyme systems, and nano-enzyme systems have already covered most of the fields of practical applications, but, although these systems have been used in all kinds of fields, they still need to be improved continuously. Single-enzyme systems are the most studied class of biosensors, but are unable to detect multiple analytes simultaneously due to enzyme specificity limitations. Detection of one or more analytes can be effectively accomplished through the utilization of multi-enzyme biosensors. The selection of enzymes plays a critical role in the advancement of biosensors designed for multi-enzymatic systems. This is due to the requirement of enzymes to have comparable operating conditions, such as temperature, pH, and concentration. In addition, the use of economical nano enzymes is a favorable technology to promote the development of biosensors. However, despite all the advantages of utilizing nano enzymes, there are still many hurdles to overcome to advance their application. These challenges include the lack of substrate specificity, possible contamination of the apoenzymes surfaces due to the uptake of dominant mixtures, and the limited range of enzyme types they can mimic. Hence, it is imperative to persistently explore the inherent active sites of enzymes to imitate and augment specificity. Furthermore, the combination or synergy between natural enzymes and nano enzymes holds potential as viable alternatives to tackle this issue effectively, as their interplay can amplify the selectivity and sensitivity of these systems.

At present, the primary challenge in enzyme electrochemical biosensors revolves around the process of immobilizing enzymes. This process directly impacts the sensor’s stability, reproducibility, and other functionalities. Despite the promising development of electrochemical biosensors in recent years, their storage and stability are still challenges that need to be addressed. In response to these issues, this paper discusses how several nanomaterials such as metals and their oxides, graphene-related materials, metal–organic frameworks, carbon nanotubes, and conductive polymers can be used as support materials to improve biosensors for the detection of various analytes. Enzyme electrochemical biosensors centered around nanomaterials represent a pivotal focus in the realm of biosensor investigation. Despite the diverse assortment of nanomaterials, which exhibit distinct chemical structures, properties, and morphologies, the favorable influence of nanomaterials in biosensors can be ascribed to three primary factors: substantial surface-area-to-volume ratios, elevated electrical conductivity, and exceptional biocompatibility. It was found that enzyme-based nanomaterials for electrochemical biosensor applications have three major effects. Firstly, they enhance the charge transfer between the enzyme and the electrode, allowing direct electron transfer, which improves the efficiency and high conductivity of the biosensor. Second, nanomaterials improve the immobilization and stabilization conditions of the enzyme, which ensures that the enzyme maintains its biocatalytic activity for a longer period. Finally, nanomaterials enhance the catalysis of electrochemical reactions, leading to faster and more efficient detection of target analytes. On the other hand, scientists are increasingly favoring the use of nanomaterials for biosensors because they are easy to synthesize and can be easily electrochemically treated directly on the electrode surface. In addition, biosensors based on nanocomposites and newly discovered nanostructures have shown promising applications. These new advances have the potential to revolutionize the field of electrochemical biosensors, enabling more accurate and sensitive detection of a wide range of analytes.

## Data Availability

Not Applicable.

## Abbreviations

List of abbreviations and acronyms.

Electrochemically Active Substances	EAS	Poly (meta-phenylenediamine)	PmPD
Flavin adenine dinucleotide	FAD	glycerol kinase	GK
Flavine adenine dinucleotide, reduced	FADH_2_	glycerol-3-phosphate oxidase	GPO
Acetaminophen	AP	chitosan	CHIT
Ascorbic acid	AA	DL-Thioctic acid	TA
Uric acid	UA	nanoporous gold	(NPG
Mediated electron transfer	MET	multi-walled carbon nanotubes-ionic liquid	MWCNTs-IL
Direct electron transfer	DET	cholesterol oxidase	CO
Glucose oxidase	GOx	Glucoamylase-displayed bacteria	GA-bacteria
Ferulic acid	FA	glucose dehydrogenase-displayed bacteria	GDH-bacteria
Graphene oxide	GO	nano enzymes	NZs
Nanoparticles	AuNP	Metal–organic frameworks	MOFs
Multi-walled carbon nanotubes	MWCNTs	superoxide dismutase	SOD
Dendritic gold nanostructures	DGNs	carbon microfibres	CFs
p-coumaric acid	p-CA	platinum microparticles	PtMPs
Vanadium dioxide	VO_2_	Histidine	His
Graphite fiber	GF	Gold nanoclusters	AuNCs
Graphite fiber electrode	GFE	reduced graphene oxide	RGO
Uric acid	UA	S-doped rGO	S-rGO
Amperometry	CPA	hydrogen peroxide	H_2_O_2_
Pulsed amperometry	PA	γ-cyclodextrin	γ-CD
differential pulse voltammetry	DPV	crosslinked MOF-919-NH_2_ nanozyme	MOF-919-NH_2_@γ-CD
Glutaraldehyde	GA	Vibrio parahaemolyticus	VP
lactate dehydrogenase	LDH	Phenylboronic acid	PBA
lactate oxidase	LOx	CuO_2_ nanodot-mediated metal–organic framework nanozymes	CP@MOF
Nicotinamide adenine dinucleotide	NAD^+^	glutathione-modified Fe_1−x_S nanoparticles	Fe_1−x_S
flow injection analysis	FIA	microflower molybdenum disulfide	MoS_2_-MF
Lactase	LAC	Fe–Mn bimetallic nanozyme	FeMnzyme
cellobiose dehydrogenase	CDH	MnO_2_ nanorods	MnO_2_ NRs
Biogenic amines	BA	Fe-Mn bimetallic nanozyme	Dex-FeMnzyme
diamine oxidase	DAO	Total antioxidant capacity	TAC
Monoamine oxidase	MAO	edge-functionalized graphene	FG
chitosan	CH	polyaniline	PANI
carbon nanofibers	CNFs	metal–organic frameworks	MOFs
horseradish peroxidase	HRP	microfibrillated cellulose	MFC
β-galactosidase	β-gal	Indian gum Tragacanth	IGT
galactose oxidase	GaOx	Ionic liquid functionalized graphene with 1-methylimidazole	G-IL
sodium dodecyl benzene sulfonate	NaDBS	carbon nanotubes	CNTs
invertase	INV		
